# NADPH Oxidases and Mitochondria in Vascular Senescence

**DOI:** 10.3390/ijms19051327

**Published:** 2018-04-29

**Authors:** Gloria Salazar

**Affiliations:** Department of Nutrition, Food and Exercise Sciences and Center for Advancing Exercise and Nutrition Research on Aging (CAENRA), Florida State University, Tallahassee, FL 32306, USA; gsalazar@fsu.edu; Tel.: +1-850-644-1220

**Keywords:** senescence, Nox1, Nox4, mitochondria, NF-κB, VSMCs, SASP, zinc, ZnT3, ZnT10

## Abstract

Aging is the major risk factor in the development of cardiovascular diseases (CVDs), including hypertension, atherosclerosis, and myocardial infarction. Oxidative stress caused by overproduction of reactive oxygen species (ROS) and/or by reduced expression of antioxidant enzymes is a major contributor to the progression of vascular senescence, pathologic remodeling of the vascular wall, and disease. Both oxidative stress and inflammation promote the development of senescence, a process by which cells stop proliferating and become dysfunctional. This review focuses on the role of the mitochondria and the nicotinamide adenine dinucleotide phosphate (NADPH) oxidases Nox1 and Nox4 in vascular senescence, and their contribution to the development of atherosclerosis. Recent findings are reviewed, supporting a critical role of the mitochondrial regulator peroxisome proliferator-activated receptor gamma (PPARγ) coactivator-1α (PGC-1α), the inflammatory gene nuclear factor κB (NF-κB), zinc, the zinc transporters (ZnTs) ZnT3 and ZnT10, and angiotensin II (Ang II) in mitochondrial function, and their role in telomere stability, which provides new mechanistic insights into a previously proposed unified theory of aging.

## 1. Senescence

**Figure 1:**
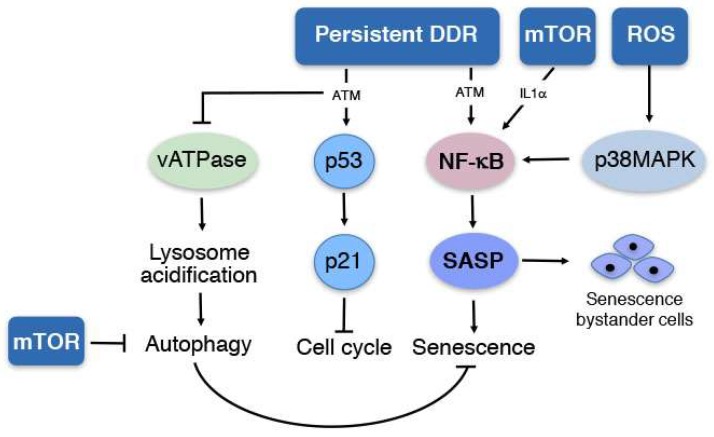
Molecular pathways involved in senescence. Persistent DNA damage response (DDR) activates the ATM/p53/p21 pathway to stop the cell cycle, while preventing lysosomal acidification that inhibits autophagy, and activating NF-κB that induces senescence associated secretory phenotype (SASP). mTOR and reactive oxygen species (ROS) can induce senescence by DDR-independent mechanisms through the p38MAPK/NF-κB/SASP pathway. mTOR can also inhibit autophagy by a NF-κB-independent mechanism to induce senescence. Altogether, cell cycle arrest, reduced autophagy, and secretion of SASP components mediate vascular dysfunction and accelerated aging.

Cellular senescence, a hallmark of mammalian aging, is a process in which cells stop proliferating and become dysfunctional, due to accumulation of mutations that cause damage to the DNA, proteins, and lipids. The reduction in proliferating cells over time impairs repair mechanisms, which are needed to cope with normal wear and tear [1]. Senescence is stimulated by a variety of stress conditions, including elevated ROS, radiation, and carcinogens. In these conditions, cell cycle arrest, mediated by upregulation of cell cycle inhibitors like p21, p53, and p16, is stimulated by activation of the DNA damage response (DDR) (Figure 1). The DDR activates stress kinases like ataxia-telangiectasia mutated (ATM) needed for the upregulation of p53. However, in many instances, DNA repair is impaired, and cells remain in a permanent state of cell cycle arrest, while retaining a metabolically active state. Senescent cells secrete an abnormal variety of molecules, including inflammatory cytokines, growth factors, ROS, and extracellular matrix components; these molecules modify the cellular microenvironment, creating a vicious cycle of oxidative stress and inflammation causing tissue dysfunction during aging. This process is known as the senescence associated secretory phenotype (SASP) [2,3]. While senescence may protect against the initiation of tumorigenesis, due to lack of proliferation, the development of the SASP may promote proliferation of an established tumor [4,5]. Furthermore, SASP components, like ROS, promote senescence in bystander cells, which contribute to the spread of senescence in organs and tissues during aging [6]. Therefore, senescent cells are considered a common target in therapeutic interventions against aging and age-related diseases, like CVD and cancer [1].

Senescent cells secret more than 40 molecules that are part of the SASP, which include inflammatory cytokines (interleukins IL6, IL8 and IL1α), growth factors (hepatocyte growth factor (HGF), fibroblast growth factor (FGF)), growth regulators, like growth-regulated oncogene (GROα), cell survival regulators, like osteoprotegerin (OPG), shed cell surface molecules, including intercellular adhesion molecule 1 (ICAM-1) and urokinase-type plasminogen activator receptor (uPAR), and metalloproteinases (MMPs) [7]. Expression of these molecules is mediated, for the most part, by stress-induced activation of signaling pathways, including NF-κB and p38 mitogen activated kinase (p38MAPK) (Figure 1) [8,9]. NF-κB is the major regulator of a large set of SASP components, including IL1α, IL6, and IL8 [7,10]. Expression of these SASP components requires persistent activation of DDR. In turn, the SASP promotes the activation of the DDR in bystander cells, which causes senescence [11]. The mammalian target of rapamycin (mTOR) and p38MAPK were also reported as major regulators of the SASP. Inhibition of mTOR by rapamycin reduces the expression of IL1α, leading to reduced transcriptional activity of NF-κB and reduced prostate tumor growth induced by senescent fibroblasts [12]. On the other hand, activation of p38MAPK induces the SASP in a DDR-independent manner in human fibroblasts, which is associated with activation of NF-κB [9].

In many cell types, the SASP is driven by IL1α [10] and by IL1β [13]. For example, in vascular smooth muscle cells (VSMCs) IL1α stimulates the secretion of IL6, IL8, monocyte chemoattractant protein-1 (MCP-1α), macrophage inflammatory proteins 1α and 1β (MIP-1α and MIP-1β), and active MMP-9 [14]. Thus, accumulation of senescent VSMCs in arteries during aging is likely the cause of pathologic vascular remodeling and arterial stiffness. Through the SASP, these senescent cells may also contribute to the spread of senescence in the adventitia and endothelium and the recruitment of inflammatory cells.

Another protective mechanism that becomes dysfunctional during aging is autophagy [15,16], a catabolic process by which protein aggregates and dysfunctional organelles, like mitochondria, are targeted to autophagosomes. Lysosomes fuse with autophagosomes, forming an autolysosome in which cargo content is degraded. A critical step in lysosomal function is acidification, a process mediated by the vacuolar ATPase (vATPase) [17]. Kang et al. [18] recently reported that the kinase, ATM, phosphorylates a subunit of the vATPase, decreasing its activity and increasing lysosomal pH (Figure 1). Impaired lysosomal acidification reduced lysosome- and autophagy-dependent degradation, leading to the accumulation of dysfunctional mitochondria.

Altogether, stress conditions, summarized in Figure 1, induce cellular senescence by activation of DDR and the downstream kinase ATM. ATM induces cell cycle arrest by upregulating p53/p21 pathway, induces the secretion of SASP components via NF-κB, and inhibits autophagy by preventing lysosomal acidification. DDR-independent pathways are mediated by mTOR- and p38MAPK-dependent regulation of the NF-κB/SASP pathway. Secreted SASP components then promote tissue dysfunction by inducing senescence in bystander cells. Additionally, DDR-independent activation of mTOR can induce senescence by inhibiting autophagy. Although many molecular pathways, such as the ones described above, have been elucidated in vitro and in vivo, interaction among molecular pathways and identification of new regulators of these pathways remain an active area of research. These pathways can be selectively activated in different tissues depending on the expression of ROS generating enzymes, such as NADPH oxidases, expression of growth factor and hormone receptors, and downstream signal transduction pathways.

## 2. NADPH Oxidases in the Vasculature

NADPH oxidase (Nox) enzymes are a family of membrane-bound complexes formed by Nox1, Nox2, Nox3, Nox4, Nox5, Duox1, and Duox2. These enzymes use NADPH as a source of electrons to generate superoxide. An overview of the structure and function of NADPH oxidases and their role in disease in the cardiovascular system can be found in recent reviews [19,20,21,22]. Nox1, Nox2, and Nox4 are expressed in the vasculature of mouse and rat, while Nox5 is expressed only in humans [23]. Nox1, Nox2, and Nox5 produce superoxide, while Nox4 produces hydrogen peroxide [24]. In addition to p22phox, which interacts with the catalytic domain of Nox1, Nox2, and Nox4 in membranes, these enzymes associate with cytosolic regulators. Nox1 and Nox2 bind to the Nox organizer 1 (NOXO1) [25], the Nox activator 1 (NOXA1) [26,27], and the small GTPase Rac1 [28], while Nox4 interacts with the polymerase-δ activating protein 2 (Poldip2) [29]. The activity of Nox1 and Nox2 is induced by the recruitment of cytosolic regulators, while Nox4 is constitutively active [30]. In contrast to other NADPH oxidases, Nox5 lacks cytosolic regulators, and contains a cytosolic domain with calcium binding sites; thus, its enzymatic activity is upregulated by calcium [31]. This review focuses on Nox1 and Nox4 and their contribution to cellular senescence mainly in VSMCs.

## 3. Angiotensin II in Mitochondrial Function, Nox1 Function, and Senescence

Many molecules involved in age-related diseases also modulate cellular senescence. Examples are those in the signaling pathways mediated by Ang II, a peptide hormone and the key effector of the renin angiotensin system. Activation of this pathway increases with age, and contributes to cellular senescence in vivo in the vascular wall [32,33], and in vitro in VSMCs [32,34].

Circulating Ang II induces hypertension while local and intracellular production of Ang II results in inflammation, cell proliferation, and fibrosis [35], as well as atherosclerosis [36]. Ang II exerts its effects by binding to angiotensin II type 1 receptor (AT_1_R) and type 2 (AT_2_R) receptors [35]. AT_1_R is abundant in the vasculature and mediates Ang II pathologic effects, including ROS production, hypertrophy, proliferation, and inflammation [37], while AT_2_R is predominant in fetal tissues, and is associated with Ang II protective effects [38,39]. Activation of AT_2_R stimulates vasodilation by increasing endothelial nitric oxide synthase (eNOS) activity, and nitric oxide (NO) production in endothelial cells [40], contributing to reduced blood pressure in spontaneously hypertensive rats [41]. The activation of eNOS by AT_2_R is mediated by increased phosphorylation of activating residues in eNOS induced by Akt, and dephosphorylation of inhibitory residues by phosphatases [42].

**Figure 2:**
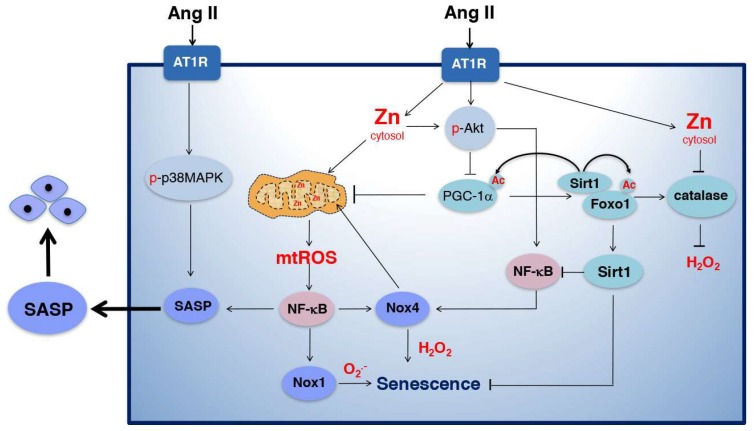
The role of Ang II in vascular senescence. Ang II binding to AT_1_R activates signal transduction pathways, including phosphorylation of p38MAPK, a driver of the SASP, and Akt, a negative regulator of PGC-1α function. Akt phosphorylates PGC-1α, increasing its acetylation, which reduces the binding of PGC-1α/FoxO1 (forkhead box O 1) to catalase and Sirt1(silence information regulator 2-like 1) promoters, reducing the expression of these target genes. Reduced Sirt1 expression further increases PGC-1α and FoxO1 acetylation, creating a negative feedback loop. Ang II also increases cytosolic zinc levels, leading to mitochondrial zinc accumulation that causes mitochondrial dysfunction and mitochondrial ROS (MitoROS) production that mediates NF-κB activation and Nox1 expression. NF-κB drives the SASP, and also mediates Nox4 upregulation and oxidative stress. Sirt1 inhibits senescence by deacetylating NF-κB, PGC-1α and FoxO1, thus improving mitochondrial function and the antioxidant capacity, while zinc induces senescence by causing mitochondrial dysfunction, increasing Nox1 and reducing catalase expression. SASP stimulates senescence in bystander cells.

In VSMCs, Ang II acts through the AT_1_R to activate the NADPH oxidase Nox1, leading to superoxide production and phosphorylation of downstream tyrosine and serine/threonine kinases [37,43]. The kinases p38MAPK, Akt, and extracellular signal-regulated kinase (ERK)1/2 mediate Ang II-induced effects in VSMCs [44,45]. We previously reported that inhibition of any of these kinases reduced senescence induced by Ang II in VSMCs [46], suggesting that Ang II-induced senescence is complex, and could be mediated by multiple mechanisms. Some of the molecular mechanisms regulating Ang II-induced senescence reported by us and others are summarized in Figure 2.

First, Ang II induces mitochondrial dysfunction by inhibiting the activity of PGC-1α. The PGC-1 family of transcriptional co-activators (PGC-1α, PGC-1β, and PRC) regulate the function of many transcription factors involved in mitochondrial biogenesis and function, including Nrf-2 (nuclear factor (erythroid-derived-2)-related factor 2) [47]. PGC-1α also co-activates transcription factors involved in the expression of antioxidant enzymes, like superoxide dismutase (SOD) 2 and Glutathione peroxidase 1 (Gpx-1) [48,49]. We demonstrated that Ang II inhibits PGC-1α activity by increasing its phosphorylation in serine 570 by Akt, which is necessary for the binding of the acetyltransferase GCN5 (general control non-repressed 5), and the acetylation and inhibition of PGC-1α in VSMCs [50]. Downregulation of PGC-1α activity by Ang II reduces the binding of the transcription factor FoxO1 to catalase promoter reducing the expression of this antioxidant enzyme, increasing ROS levels and causing hypertrophy of VSMCs [50]. We also demonstrated that downregulation of catalase expression by siRNA increases ROS levels and induces senescence in VSMCs [34]. Additionally, Ang II also causes mitochondrial dysfunction by increasing superoxide production via the electron transfer chain (ETC). Inhibition of complex I, complex II, or treatment with the mitochondrial ROS scavenger, MitoTEMPO (mitochondrial 2,2,6,6-tetramethyl-piperidine-1-oxyl), reduced superoxide levels and senescence in VSMCs in response to Ang II [51]. Ang II also affects mitochondrial membrane potential by mediating the opening of the mitochondrial K_ATP_ channel, resulting in superoxide production that was required for activation of MAP kinases [52] and autophagy [53]. Moreover, upregulation of NADPH oxidase activity by Ang II was also involved in mitochondrial dysfunction in endothelial cells [54]. ROS generated by NADPH oxidases react with NO, producing peroxynitrate, which promotes mitochondrial dysfunction, measured by reduced mitochondrial membrane potential, respiration, and glutathione levels, and increased superoxide production [54].

Silence information regulator 2-like 1 (Sirt1), a member of the NAD-dependent sirtuin family of histone deacetylases, increases mitochondrial biogenesis and the expression of antioxidant enzymes by deacetylating and activating PGC-1α [55] and FoxO transcription factors [56]. Sirt1 is activated by calorie restriction, and extends lifespan in many organisms, including worms [57], flies [58], and mice [59]. We demonstrated that phosphorylation and acetylation of PGC-1α and FoxO1 by Ang II reduces Sirt1 transcription and catalase expression, leading to senescence of VSMCs [60]. Downregulation of either PGC-1α, FoxO1, or Sirt1 by siRNA induces senescence of VSMCs in the absence of Ang II, while genetic deficiency of PGC-1α induces senescence in aortas of mice in vivo [60]. In addition to mitochondrial dysfunction and fragmentation, PGC-1α deficiency also reduces the activity of telomerase, an enzyme that extends telomeres, and increases DNA damage, leading to telomere attrition and replicative senescence [60,61]. Overexpression of Sirt1 reduces PGC-1α and FoxO1 acetylation, inhibiting Ang II-induced senescence [60]. Furthermore, we showed that FoxO1 binds to the Sirt1 promoter, increasing its transcription [62]. Overall, Ang II mediates senescence by increasing PGC-1α acetylation, reducing FoxO1 binding to catalase and Sirt1 promoters, which increases ROS levels and reduces Sirt1-induced deacetylation of PGC-1α and FoxO1, creating a negative feedback loop that causes senescence.

**Figure 3:**
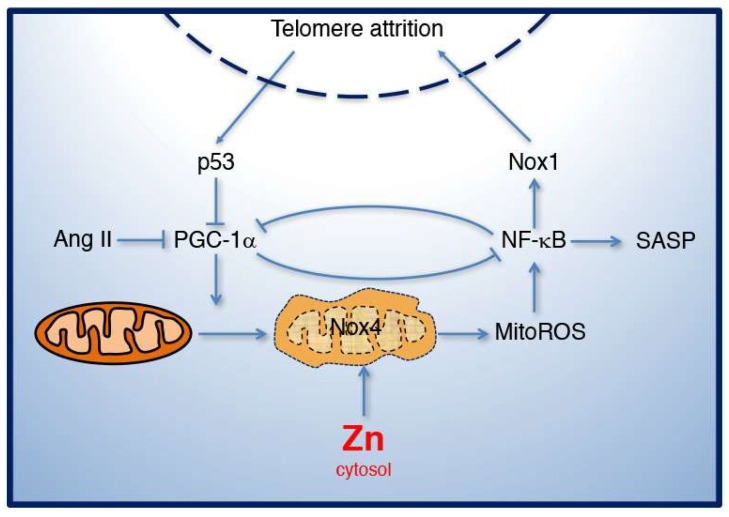
PGC-1α and Nox1 in telomere and mitochondrial dysfunction during aging. Telomere dysfunction induces mitochondrial dysfunction through the p53-dependent downregulation of PGC-1α. Dysfunctional mitochondria produce mitochondrial ROS (MitoROS) that activates NF-κB-dependent upregulation of Nox1, causing telomere attrition and replicative senescence. Ang II induces senescence by inhibiting PGC-1α function, while zinc overload causes senescence by increasing MitoROS and Nox1 expression. In conditions of stress, Nox4 localizes in the mitochondria, also contributing to MitoROS production.

Second, Ang II induces inflammation by activating NF-κB [39]. Once activated, NF-κB translocates to the nucleus, increasing the expression of pro-inflammatory cytokines, including IL6, IL1β, and tumor necrosis factor-alpha (TNF-α), chemokines such as MCP-1, and cell adhesion molecules, such as vascular cell adhesion molecule-1 (VCAM-1) and ICAM-1 [35,63]. Increased production of these pro-inflammatory molecules through SASP contributes to vascular injury and atherosclerosis. In the heart, NF-κB interacts with the promoter of PGC-1α, reducing its transcription [64], suggesting that NF-κB could also mediate the downregulation of PGC-1α in response to Ang II in VSMCs. Interestingly, PGC-1α overexpression reduced NF-κB transcriptional activity and the levels of IL6 and TNFα in skeletal muscle, in vitro [65] and reduced the pro-inflammatory cytokine IL12 in skeletal muscle, in vivo [66]. Similarly, in VSMCs and endothelial cells, PGC-1α overexpression reduced NF-κB activation mediated by TNFα [67]. These data suggest that PGC-1α may reduce inflammation by inhibiting NF-κB and the associated SASP response (Figure 3).

Third, intracellular ROS produced by AT_1_R activation exacerbates inflammatory responses by acting as a second messenger in signal transduction pathways [68]. The increase in ROS levels is attributed, in part, to downregulation of antioxidant enzymes, as mentioned before, and also to upregulation of NADPH oxidases, which contribute to vascular dysfunction and CVD [69]. We demonstrated that Ang II-induced senescence is a zinc-dependent mechanism in VSMCs [34]. Chelation of zinc with TPEN reduces Ang II-induced senescence, while zinc overload, in the absence of Ang II, increases NADPH oxidase activity, ROS levels, and senescence [34]. We also demonstrated that zinc- and Ang II-induced senescence is mediated by upregulation of Nox1 expression [46,70] and that Nox1 overexpression is sufficient to induce VSMC senescence [46]. Nox1-overexpressing cells show reduced proliferation and expression of telomerase, and increased DNA damage, measured by increased phosphorylation of the histone variant H2AX (γH2AX), suggestive of replicative senescence due to telomere attrition. Further, we showed that upregulation of Nox1 expression by zinc overload is mediated by mitochondrial ROS-dependent activation of NF-κB [70]. Observations by Katsuyama et al. also support a role for mitochondria in Nox1 expression in VSMCs. Inhibitors of mitochondrial oxidative phosphorylation, like oligomycin, increase Nox1 expression in response to PDGF. Mechanistically, alteration of the ETC upregulates the activating transcription factor 1 (ATF1) activity increasing Nox1 transcription [71]. The role of Nox1 and mitochondria in Ang II-induced senescence was also shown by Tsai et al. This report shows that Nox1 is required for PGC-1α phosphorylation leading to reduced mitochondrial function and mitochondrial ROS production that mediates senescence in response to Ang II in VSMCs [72]. Thus, it is possible that two waves of ROS production by Nox1 are required for the development of senescence, an early activation of Nox1 that stimulates signal transduction and phosphorylation of PGC-1α, causing mitochondrial dysfunction and a second wave mediated by upregulation of Nox1 expression, which requires mitochondrial ROS. This model is consistent with our data showing that ROS levels are upregulated by Ang II or zinc after only 30 min of treatment, and that zinc promotes a robust early upregulation of NADPH oxidase activity [34].

Nox1 activity is upregulated by cytosolic components, like Rac1, as mentioned before. Interestingly, overexpression of Rac1 was sufficient to induce senescence of mouse embryonic fibroblasts (MEFs), which was associated with increased ROS levels, genomic instability, and DNA damage [73]. Rac1 was also upregulated during hypoxia-induced senescence in endothelial cells [74]. Overexpression of a dominant negative Rac1 mutant reduced the effect of hypoxia in senescence [74], suggesting that in these cells, Rac1 expression could be a critical regulator of senescence. Constitutive activation of Rac1, achieved by overexpression of a dominant active Rac1 mutant (Rac1 V12), was associated with mitochondrial dysfunction and senescence in endothelial cells [75]. The Rac1 V12 mutant induced ceramide production, leading to upregulation of mitochondrial ROS that promoted stress-induced premature senescence (SIPS), but not replicative senescence [75]. Since ceramide, a lipid that act as a second messenger, causes mitochondrial ROS production by disrupting the ETC [76], it is possible that Rac1 causes senescence by NADPH oxidase-dependent and -independent mechanisms.

Upregulation of Nox1 in VSMCs not only affects the function of these cells, but also regulates endothelial function. The role of NADPH oxidases in VSMCs in endothelial function was shown using a transgenic mouse overexpressing Nox1 in VSMCs. Transgenic animals infused with Ang II show increased production of superoxide and hydrogen peroxide compared with C57Bl/6 in the same conditions, which was associated with eNOS uncoupling, reduced NO production, and altered vasodilation [77]. Ang II also acts on endothelial cells causing senescence [78]. Similar to VSMCs, Ang II-induced senescence in endothelial cells was mediated by Akt activation and depolarization of mitochondrial membrane potential [78]. These findings suggest that Akt-mediated PGC-1α inactivation could be also involved in senescence in these cells.

Altogether, Ang II is a powerful inducer of cellular senescence by increasing Nox1 function, decreasing PGC-1α and mitochondrial function, antioxidant capacity, and by increasing the activity of two major drivers of SASP, p38MAPK and NF-κB. The significance of the Ang II/AT_1_R axis in aging was demonstrated by the fact that AT_1_R gene deficiency promotes longevity in mice [79].

## 4. Mitochondrial Dysfunction and Zinc Homeostasis in Aging

Increased mitochondrial ROS observed during aging is viewed as both a cause and a consequence of cellular senescence. For example, mitochondrial superoxide production causing genomic and mitochondrial DNA damage increases with replicative age [80]. Replicative senescence is delayed by reduction of mitochondrial ROS by mild mitochondrial uncoupling [80]. Connective tissue-specific SOD2^−/−^ mice show accelerated aging phenotypes, short lifespan, and increased expression of the senescence marker p16^INK4A^ [81]. On the other hand, DDR-dependent activation of p21 induces senescence with delayed mitochondrial dysfunction and ROS production [82], suggesting that these events are a consequence of senescence. However, data published in the last few years suggest that telomere function and mitochondria are part of a regulatory network regulated by PGC-1α (Figure 3). Sahin et al. demonstrated that telomere dysfunction causes mitochondrial dysfunction by p53-dependent downregulation of PGC-1α and PGC-1β, leading to mitochondrial dysfunction and increased ROS production [83]. This evidence supports a unified view of aging, in which DNA damage to telomeres induces metabolic and mitochondrial compromise, leading to accelerated aging [84,85]. PGC-1α seems to be central to this regulatory network, as shown by Xiong et al. These authors demonstrated that PGC-1α gene deficiency reduces telomerase activity and telomere length, and increases DNA damage, suggesting that altered mitochondrial function causes telomere attrition. The effects of PGC-1α deficiency were reduced by the antioxidant NAC [61]. Since scavenging of mitochondrial ROS was not tested in this report, it is unknown whether mitochondria and/or NADPH oxidases are the source of ROS. Our published data provide additional mechanistic insights into this unified view of the theory of aging (Figure 3). We showed that mitochondrial ROS activates NF-κB, upregulating Nox1 expression, which reduces telomerase expression and promotes DNA damage and replicative senescence [70]. Thus, this evidence is consistent with a model in which telomere attrition causes mitochondrial dysfunction, mediated by downregulation of PGC-1α, while mitochondrial ROS causes telomere attrition, mediated by the upregulation of Nox1 (Figure 3).

Mitochondrial dysfunction is also caused by altered zinc distribution and intracellular levels, suggesting that zinc may interact with this regulatory network by regulating mitochondrial function (Figure 3). In fact, elevated cytosolic zinc causes senescence in VSMCs [34] and in endothelial cells [86]. We showed that VSMCs express eight out of the ten members of the ZnT/SLC30A (zinc transporter/solute carrier 30A) family of zinc transporters that are in charge of decreasing cytosolic zinc by transporting zinc out of the cell or into various intracellular compartments. Out of the eight ZnTs, Ang II downregulates the expression of ZnT3 and ZnT10, resulting in reduced accumulation of zinc in intracellular compartments [34]. We demonstrated that downregulation of ZnT3 or ZnT10 induces senescence, while their overexpression inhibits senescence induced by Ang II in VSMCs [34]. We also reported that these zinc transporters form homo and heterodimers involved in the accumulation of zinc in endosomes and lysosomes, and were involved in MEK/ERK1/2 signaling pathway activated by the epidermal growth factor receptor in HEK293 cells [87]. More recently, we demonstrated that elevated cytosolic zinc promoted zinc accumulation in the mitochondria, leading to upregulation of Nox1 expression in VSMCs [70]. Similarly, Nox1 expression was also elevated in VSMCs isolated from aortas of ZnT3^−/−^ mice [70], suggesting that altered intracellular zinc distribution may contribute to this effect.

In endothelial cells, zinc-induced senescence was associated with downregulation of ZnT10 and ZnT5, found at the Golgi complex, and upregulation of ZnT1 and metallothionein (MT) genes [86]. MTs are metal binding proteins, whose expression is upregulated in response to elevated cytosolic zinc [88], a mechanism that protects cells from zinc toxicity. The metal-responsive transcription factor (MTF1) is activated in response to zinc, increasing the expression of genes involved in zinc detoxification, including MTs and ZnT1, a zinc exporter found at the plasma membrane [89]. Interestingly, Zip6, a member of the Zip (Zrt- and Irt-like protein)/SLC39A family of zinc importers, was upregulated in senescent endothelial cells in response to zinc [86]. The Zip family increases cytosolic zinc by transporting zinc inside cells from the extracellular media and from intracellular compartments. Altogether, these data suggest that altered zinc levels and distribution by changes in the expression of zinc regulators, like ZnTs, Zips, and MTs, contribute to oxidative stress in the vasculature. However, it is unknown how downregulation of one ZnT, like ZnT3, could cause a change in zinc distribution in VSMCs if these cells also express other members of this family. Since ZnT3 forms heterodimers with other ZnTs [87,90] including the ones found in VSMCs, like ZnT2, ZnT4, and ZnT10 [34], it is likely that downregulation of ZnT3 may affect zinc levels more globally. Although heterodimers formed by ZnT3 and ZnT10 are active in transporting zinc [87], it is unknown whether heterodimerization is the preferred state of these transporters in vivo.

It is unknown how zinc alters mitochondrial function in VSMCs. A recent review by Nam et al. [91] summarized the role of metals, including zinc, in mitochondrial function in neurons. In these cells, accumulation of zinc in mitochondria alters mitochondrial membrane potential, reduces oxygen consumption, and increases ROS production [92,93,94,95]. Further, in HeLa cells, hypoxia causes an early accumulation of zinc (zinc wave) in the mitochondria that was required for later increases in ROS levels [96]. Interestingly, in a model of neurodegeneration induced by oxygen glucose deprivation, increased zinc levels originated from MT-III in CA1 pyramidal neurons [97], suggesting that MT may serve as a source of zinc during stress conditions. In fact, zinc is ejected from metalloproteins, including MTs, during oxidative stress, due to oxidation of SH thiols in cysteine residues [98]. Thus, ROS production by zinc accumulation in the mitochondria could mediate zinc ejection from mitochondrial MTs, creating a vicious cycle of elevated zinc and ROS production. Thus, it is anticipated that zinc may cause similar effects in mitochondria in VSMCs.

It is unknown whether altered zinc homeostasis may inhibit PGC-1α function. However, the upregulation of Akt phosphorylation in response to zinc observed by us [34] and others [99,100,101] suggest that this may be the case. The role of PGC-1α in zinc homeostasis remains unexplored.

## 5. Nox4 in Senescence

In addition to Nox1, aortic VSMCs also express Nox4 [102]; however, the role of Nox4 in senescence is controversial, since both increased and reduced expression of this enzyme causes senescence. Downregulation of Nox4 accelerates senescence in human VSMCs, which is independent of DDR activation and correlates with increased secretion of pro-inflammatory cytokines and activation of hypoxia inducible factor 1 alpha (HIF-1α) [103]. By contrast, in endothelial cells, Nox4 downregulation inhibits replicative senescence by reducing DNA damage, which is independent of telomere attrition [104]. The mechanism by which Nox4 promotes senescence in endothelial cells involves mitochondrial dysfunction. In fact, Nox4 was found to target complex I of the ETC, increasing mitochondrial ROS production that mediates senescence [105] (Figure 2).

On the other hand, upregulation of Nox4 expression in conditions of stress or during aging induces SIPS. For example, upregulation of Nox4 by ionizing radiation induces SIPS in MEFs. Nox4-derived ROS production was associated with increased migration of monocytes, supporting a role of ROS in the recruitment of inflammatory cells into irradiated tissues [106]. In endothelial cells, upregulation of Nox4 during aging causes endothelial dysfunction mediated by endoplasmic reticulum (ER) stress, leading to eNOS uncoupling [107]. Nox4-induced senescence has been reported, also in other cell types. For example, oncogenic H-Ras upregulates Nox4 expression, inducing DNA damage and senescence in human thyrocytes [108]. In human lung cells, oncogenic Ras stimulates ERK1/2 signaling, upregulating Nox1 in primary fibroblast and Nox4 in primary human embryonic fibroblast. In both cell types, Nox isoforms activate DDR and p38MAPK signaling, promoting cellular senescence [109].

Upregulation of Nox4 in conditions of stress is mediated by diverse cellular pathways, depending on the stimulus. In a p53-induced senescence model, p53 upregulates p21 to mediate cell cycle arrest, while inducing Akt activation lead to Nox4 expression and increased ROS levels [110]. Similarly, in oncogene Ras-induced senescence, Akt stimulates NF-κB-dependent upregulation of Nox4 transcription, causing oxidative stress that causes senescence [110]. This report also demonstrated that cell cycle arrest and oxidative stress are mediated by p53 through different molecular mechanisms. Interestingly, upregulation of p21 induced by p53 mediates cell cycle arrest which is associated with quiescence, but not senescence, while upregulation of Nox4 by a p53/Akt pathway is needed for the induction of cellular senescence.

Consistent with a role of Nox4 in senescence and oxidative stress in the cardiovascular system, increased expression of Nox4 and novel short isoforms of this oxidase were found in samples of failing hearts of human patients [111]. It is unknown, however, whether these novel isoforms are functional, and the type of ROS they can produce.

**Figure 4:**
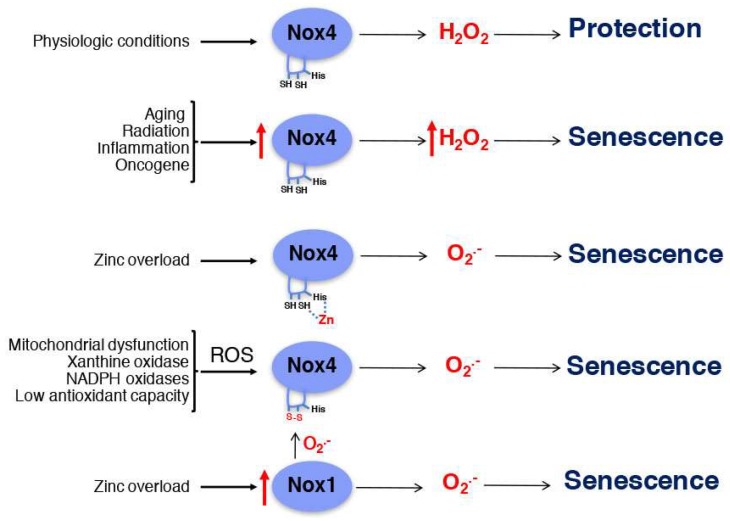
Proposed role of Nox1 and Nox4 in senescence. Hydrogen peroxide associated with basal Nox4 activity in physiological conditions protects from senescence, while overproduction of hydrogen peroxide during aging and stress conditions may promote senescence. Binding of zinc to the E-loop of Nox4 or oxidation of cysteine residues in the E-loop in response to stress conditions by overexpression of Nox1 could change the function of Nox4. Altered production of superoxide by Nox4 could mediate senescence in conditions of stress. Red arrows indicate increased expression.

Altogether, published data support both protective and disease-promoting effects of Nox4 in the cardiovascular system. The cause of these contradictory effects is unknown. It is possible that the differential effects of Nox4 in response to different stimulus and cell types are mediated by different isoforms, by altered subcellular localization, and/or by differential interaction with regulatory adaptors. It is well established that Nox4, as a constitutive enzyme, produces hydrogen peroxide, which could mediate Nox4 protective effects in physiological conditions (Figure 4). Upregulation of Nox4 expression during aging or by stress conditions, such as radiation, inflammation, and oncogenes, could mediate senescence, due to excessive production of hydrogen peroxide. However, some reports also show that Nox4 is capable of producing superoxide [112]. In fact, in the kidney, Massey et al. demonstrated that Ang II increases superoxide production by upregulating Nox4 [113], suggesting that, in stress conditions, Nox4 can produce superoxide instead of hydrogen peroxide.

The ability of Nox4 to produce hydrogen peroxide is mediated by the extracellular E-loop of the enzyme. Deletion of the E-loop or mutagenesis of two critical cysteine residues (Cys 226 and Cys 270) in this domain change the activity of Nox4 from a hydrogen peroxide- to a superoxide-producing enzyme [114]. Further, treatment with reducing agents upregulates Nox4 activity, suggesting that reduction of Cys 226 and Cys 270 in the E-loop may promote hydrogen peroxide production [115]. However, the levels of hydrogen peroxide and superoxide in reduced and oxidized conditions were not determined in this study.

In addition to cysteine residues, a conserved histidine residue (His 222) in the E-loop is also required for Nox4 activity [114]. Mutagenesis of His 222 mediates superoxide instead of hydrogen peroxide production by Nox4 [114]. Histidine and cysteine residues are involved in the interaction with zinc, for example, in zinc fingers found in transcription factors [116]. Therefore, it is possible that zinc interacting with cysteine and histidine residues in the E-loop may induce oxidative stress by changing the type of ROS produced by Nox4. This mechanism could be of importance in conditions of zinc overload, in which binding of zinc to the E-loop could promote Nox4-dependent superoxide production. Also, upregulation of Nox1 expression and superoxide production by zinc overload could also affect the oxidation state of cysteine residue in the E-loop, as illustrated in Figure 4. ROS produced by other sources in conditions of stress, such as mitochondrial dysfunction, xanthine oxidase, other NADPH oxidases, or by reduced antioxidant capacity, could potentially affect the oxidation state of the E-loop and Nox4 function. On the other hand, protein–protein interactions may protect the E-loop from oxidation, upregulating Nox4 activity and hydrogen peroxide production. For example, Poldip2 associates with Nox4 in focal adhesion, increasing its enzymatic activity and stress fiber formation during migration in VSMCs [29]. Thus, it is possible that Nox4 protective effects in senescence are mediated by hydrogen peroxide, while Nox4 deleterious effects are mediated by superoxide production. The role of zinc in Nox4 function remains unexplored.

## 6. Nox2 and Nox5 in Senescence

Although this review focuses mainly on Nox1 and Nox4, other NADPH oxidases found in the vasculature are also involved in vascular senescence, like Nox2 and Nox5. Nox2 mediated senescence in response to doxorubicin, a drug used in chemotherapy, in endothelial progenitor cells [117]. Nox2 was also involved in diabetic retinopathy by upregulating the expression of arginase [118]. This enzyme degrades arginine, the substrate of NO synthase needed for the production of NO. Thus, ROS produced by Nox2 reduced NO production, inducing senescence of retinal endothelial cells [118]. Nox2 is also upregulated during aging in aortas of mice, which was associated with impaired endothelial-dependent vasorelaxation, an effect that was reduced in Nox2^−/−^ mice [119]. Further, senescence induced by high glucose and insulin in endothelial cells was inhibited in Nox2^−/−^ endothelial cells [119]. Interestingly, resveratrol, an activator of Sirt1, promoted senescence in lung cancer cells by upregulating Nox5, but not Nox1 or Nox2 expression. The effect of Nox5 was mediated by ROS-dependent DNA damage [120]. In a study using endothelial progenitor cells to explore the role of Nox1-5 in cell senescence induced by Ang II, Nox2 and Nox4 were identified as the major contributors to senescence, while Nox1, Nox3, and Nox5 were not involved in this process [121]. Thus, the contribution of Nox1, Nox2, Nox4, and Nox5 to cellular senescence depends on the cell type and stimulus used to induce senescence.

## 7. Senescence in Atherosclerosis

Senescent vascular cells in culture present similar changes to the ones observed in aged arteries, such as increase in ROS levels in both the endothelium and VSMCs [122]. Senescent VSMCs positive for the senescence marker SA-β-gal (senescence associated-β-galactosidase) have been found in atherosclerotic plaques [123], indicating that cellular senescence could contribute to in vivo vascular dysfunction [32,124,125]. In fact, senescent VSMCs are found in the fibrous cap of human plaque [14]. Secretion of SASP components by these cells induces a state of inflammation, reduced level of extracellular components, such as collagen, causing plaque instability and rupture [14]. Further, secretion of MCP-1 by senescent VSMCs increases chemotaxis of mononuclear cells, including monocytes and lymphocytes. Thus, VSMCs are critical for plaque stability, while senescent VSMCs promote inflammation and plaque rupture [14]. Additionally, senescent VSMCs in human plaques show telomere attrition, due to downregulation of the telomeric repeat-binding factor 2 (TRF2), a critical regulator of telomere length [126]. A transgenic mouse overexpressing TRF2 in VSMCs showed more stable plaques characterized by increased fibrous cap and reduced necrotic core [126]. Thus, senescent VSMCs contribute to plaque instability through SASP- and telomere-dependent mechanisms.

In addition to senescent VSMCs, Childs et al. [127] demonstrated that plaque also contains senescent endothelial cells and macrophages. Importantly, these senescent cells accumulate in plaques of low-density lipoprotein receptor (LDLR) ^−/−^ mice that were fed a high fat diet, but not in normal adjacent aorta tissue. Furthermore, these investigators provided the most convincing evidence published so far supporting the contributing role of senescence in atherosclerosis. They generated a mouse model expressing the herpes simplex virus thymidine kinase under the control of the p16 promoter. This approach selectively kills p16 positive senescent cells in vivo. Clearance of senescent cells, including VSMCs, endothelial cells and macrophages, reduced, by 60%, plaque accumulation in the aorta tissue in response to the high fat diet [127]. This effect was independent of the level of circulating cholesterol, HDL (high-density lipoprotein), or triglycerides, suggesting that therapeutic strategies to remove senescent cells, in combination with cholesterol-reducing drugs, could be more effective in the treatment of atherosclerosis.

## 8. Nox1 and Nox4 in Atherosclerosis

ROS generated by NADPH oxidases contributes to normal vascular function, however, excessive ROS production by these enzymes mediate oxidative stress associated with increased inflammation and disease. Thus, too little or too much expression of these enzymes have been associated with vascular disease. Thus, the role of these enzymes in CVD, in particular in atherosclerosis, remains controversial.

Nox1 deficient mice showed reduced hypertension in response to Ang II [128], and reduced plaque accumulation in ApoE^−/−^ mice fed a high fat diet [129]. Similarly, Nox1 deficiency reduced plaque accumulation in ApoE^−/−^ mice treated with streptozotocin, a drug that induces type 1 diabetes mellitus [130]. The protective effect of Nox1 deficiency was also observed in a mouse model of accelerated atherosclerosis induced by partial carotid ligation in C57Bl/6 mice fed high fat diet [131]. In another report, deletion of Nox1 in ApoE^−/−^ mice showed less stable plaque, increased plasma lipids, but no differences in plaque area after 14 weeks of a Western diet, suggestive of a protective role of Nox1 in atherosclerosis [132].

In ApoE^−/−^ mice, overexpression of Nox4 in endothelial cells reduced atherosclerosis, mainly in the abdominal section of the descending aorta, in animals fed a Western diet for 24 weeks [133]. This protective effect was mediated by upregulation of T regulatory cells, which are known to protect from atherosclerosis [134], and by downregulation of T effector cells, which are involved in inflammatory responses [135]. The protective role of Nox4 in atherosclerosis has been also demonstrated in Nox4^−/−^ animal models. Genetic deletion of Nox4 in ApoE^−/−^ mice increased atherosclerosis after 20 weeks of diabetes mellitus induction, while deletion of Nox1 in the same conditions reduced atherosclerosis [136]. The protective role of Nox4 in atherosclerosis was also reported using LDLR^−/−^Nox4^−/−^ mice fed a high fat diet for 20 weeks [137]. Further, higher levels of plaque were also found in aortas of ApoE^−/−^Nox4^−/−^ mice in normal chow diet, and in partially ligated carotid arteries of those animals in response to a high fat diet [138]. Moreover, in VSMC cells, Nox4 showed a protective effect against proliferation and fibrosis, thus, maintaining the differentiated contractile phenotype of VSMCs [139].

In contrast to the protective effect of Nox4 overexpression in endothelial cells [133], overexpression of Nox4 in VSMCs was associated with increased plaque instability and rupture [140]. Further, Nox4 was upregulated in VSMCs in atherosclerotic lesions compared with media VSMCs, which was associated with increased senescence and apoptosis in those cells [140]. With respect to the role of aging in Nox4 expression, Vendrov et al. found that Nox4 was upregulated in the vasculature of old mice, compared with young animals, and in aortic VSMCs derived from samples of old, compared with young human subjects [141]. Increased Nox4 expression was also correlated with atherosclerosis severity in human subjects [141]. More recently Lozhkin et al. reported that upregulation of Nox4 in VSMCs also contributed to inflammation during aging and in atherosclerosis [142]. Interestingly, upregulation of inflammatory genes, including IL6, a component of the SASP, was observed in atherosclerotic arteries from ApoE^−/−^ mice, as well as from human carotid arteries. [142].

Reports in the literature support the idea that Nox4 is protective, while Nox1 is deleterious for atherosclerosis development. As mentioned before, the differential effect of Nox1 and Nox4 could be associated with the type of ROS produced by these enzymes and/or the place where ROS is produced. Nox1 produces superoxide and localizes in the plasma membrane, caveolae, and endosomes [102,143], while Nox4 produces hydrogen peroxide and localizes in the nucleus, focal adhesions, and the ER [102]. However, during aging and in disease conditions, such as diabetes and cardiac hypertrophy, Nox4 is found in the mitochondria, which causes mitochondrial oxidative stress. The upregulation of Nox4 in the mitochondria was observed in a model of diabetes in rat, mainly in the cortex of kidney cells [144]. In mesangial cells of the kidney, Nox4 was enriched in purified mitochondria and colocalized with the mitochondrial marker MitoTracker. Further, downregulation of Nox4 in these cells reduced mitochondrial ROS production in response to glucose [144]. Nox4 was also upregulated in the mitochondria in the heart under hypertrophic conditions, including Ang II, transverse aortic constriction, and phenylephrine, which was associated with increased mitochondrial superoxide production [145]. Nox4 was also found in mitochondria in aged arteries in hyperlipidemic conditions, contributing to mitochondrial ROS production [141]. Thus, localization of Nox4 in the mitochondria in conditions of stress may promote mitochondrial oxidative stress that may induce senescence by a Nox1-dependent mechanism, as shown in Figure 4, and may also contribute to accelerated atherosclerosis in conditions of stress. It is unknown whether altered subcellular localization in conditions of stress, together with oxidation of cysteine residues in the E-loop and/or interaction with unknown adaptors, may promote Nox4-mediated superoxide production during atherosclerosis development.

## 9. Concluding Remarks

In summary, PGC-1α, the NADPH oxidases Nox1 and Nox4, and mitochondria, work together in a regulatory network to maintain mitochondrial function and telomere stability. Based on published data by us and others, we propose that telomere dysfunction mediated by PGC-1α deficiency is mediated by the upregulation of Nox1. Mechanistically, increased mitochondrial ROS produced by PGC-1α deficiency activates NF-κB, increasing Nox1 expression, which reduces telomerase expression, and increases DNA damage and replicative senescence. Based on this model, either p53 activation, PGC-1α inhibition, dysfunctional mitochondria, or altered zinc levels or distribution should promote SASP and induce senescence in bystander cells. We propose that therapeutic interventions targeting PGC-1α and NF-κB should provide better protection against age-related diseases, such as atherosclerosis.

## Abbreviations

Ang II AT_1_R AT_2_R	angiotensin II angiotensin II type 1 receptor angiotensin II type 2 receptor
ATF1	activating transcription factor 1
ATM	ataxia-telangiectasia mutated
CVD	cardiovascular disease
DDR	DNA damage response
eNOS ERK ER	endothelial nitric oxide synthase endoplasmic reticulum extracellular signal-regulated kinase
FGF	fibroblast growth factor
FoxO	forkhead box O
GCN5	general control non-repressed protein 5
Gpx	glutathione peroxidase
GRO HDL	growth-regulated oncogene high-density lipoprotein
HIF-1α	hypoxia inducible factor 1 alpha
HGF	hepatocyte growth factor
ICAM-1	intercellular adhesion molecule 1
IL LDL	Interleukin Low-density lipoprotein
MEF	mouse embryonic fibroblast
MIP-1 MitoTEMPO	macrophage inflammatory proteins 1 mitochondrial 2,2,6,6-tetramethyl-piperidine-1-oxyl
MMP	metalloproteinase
MCP-1 MT MTF1	monocyte chemoattractant protein-1 metallothionein metal-responsive transcription factor 1
mTOR NADPH	mammalian target of rapamycin nicotinamide adenine dinucleotide phosphate
NF-κB	nuclear factor κB
NO	nitric oxide
NOXA1	Nox activator 1
NOXO1	Nox organizer 1
Nrf-2	nuclear factor (erythroid-derived-2)-related factor 2
OPG p38MAPK	Osteoprotegerin p38 mitogen activated kinase
PGC-1α γH2AX	peroxisome proliferator-activated receptor γ (PPARγ) coactivator-1α phosphorylated H2AX
Poldip2	polymerase-δ activating protein 2
ROS	reactive oxygen species
SA-β-gal	senescence associated β galactosidase
SASP	senescence associated secretory phenotype
SIPS SLC	stress induced premature senescence solute carrier
Sirt1	silence information regulator 2-like 1
SOD	superoxide dismutase
TNF-α	tumor necrosis factor-alpha
TRF2	telomeric repeat-binding factor 2
uPAR	urokinase-type plasminogen activator receptor
vATPase	vacuolar ATPase
VCAM-1	vascular cell adhesion molecule 1
VSMCs Zip	vascular smooth muscle cells Zrt- and Irt-like Protein
